# Foreign

**Published:** 1841-12-25

**Authors:** 


### FOREIGN.

*Quackery and Humbug.—Inflammation.*—The Medical Society of London held its first meeting for the session on the evening of September 27, 1841; Dr. Clutterbuck, president, in the chair. There was a very full attendance of members and visitors. In some preliminary observations, Dr. Clutterbuck took occasion to allude to several kinds of "quackery" and "humbug" which have lately prevailed, to a certain extent, in the profession. He particularly alluded to some new operations for defective vision, to operations for stammering, and to mesmerism. He classed the three together as being equally worthy of condemnation. He could not help expressing regret and surprise, that such proceedings as those should obtain even a transient notoriety in a profession so strictly one of fact as that of medicine. When, however, they did, unhappily for the public and the profession, succeed in obtaining dupes, then it was the duty of societies, like the Medical Society of London, to expose and condemn the fallacies. This was one great benefit which medical societies conferred upon the public.

After some further observations on the mode of conducting proceedings in the society, a paper was read from the pen of the president, entitled, an "Attempt to show that most Diseases originate, directly or indirectly, in Inflammation." The paper was long and elaborate, and we can scarcely venture upon an abstract of it in the present Lancet. Suffice it to say, that by a course of very ingenious reasoning, the author endeavoured to show, that almost all diseases, except those which might be considered as merely symptoms, had their origin, more or less remotely, in that state which is denominated inflammation. This inflammation might be of such a character as to be at once recognized, or it might be so insidious as to destroy life, before its presence was detected. Happily, however, the practitioner had, most generally, sufficient evidence of its presence; for, although the four more ordinary signs of inflammation—heat, redness, swelling, and pain—were not to be detected in all cases of inflammation of the vital organs, still, when these were inflamed, there was such an exaltation of the vital properties, so general a febrile state, such an interference with the function of the organ, and such evidence of sympathy manifested by other and remote organs, that the diagnosis was rendered less difficult than it otherwise would be. The author proceeded to enumerate various diseases which originated or were accompanied by inflammation; and concluded by observing, that out of the one hundred and fifty genera enumerated in the nosology of Cullen, one-half of them were merely symptoms or varieties of disease, and not diseases themselves. Of the remaining genera, another half had their origin in inflammation.

*Lancet.*

---

*Cases of Granular Disease of the Kidney.*— John Fisher, aged 31, a milkman, was admitted into Sutherland ward, King's College Hospital, on the 19th of April, under the care of Dr. Todd. He is an intemperate man, drinking freely of porter and gin. He was quite healthy until six months ago, when he was much exposed to cold and wet, and caught a violent cold. He had cough, with grayish expectoration, great dyspnœa, and palpitation, which was much increased by exertion, and which soon compelled him to keep to his bed. He says that at this time he had inflammation of the chest, the chief symptoms being great pain in the right side of that region. He kept his bed for six weeks, and at the end of that time first observed that his body was puffed in various parts. He noticed the swelling first in the face, then in the feet and ankles; the abdomen became swollen at the same time. During the six weeks mentioned, the bowels were much affected, and he had severe pain and purging of a black-looking fœtid matter. For the last two or three months he has been complaining of dyspnœa, and a slight cough, with mucous expectoration; occasional palpitation of the heart; the legs have been more or less œdematous, and the abdomen swollen. The urine has been rather scanty, and he has passed it frequently. At present there is great œdema of the feet and legs, and ascites; the face and eyelids are puffed. He has dyspnœa and frequent cough, attended with frothy expectoration; pulse 100, feeble; skin dry. The chest is resonant on percussion; rather less so over the inferior part of both sides behind; respiration pure in front; some large crepitations behind at the inferior part. No impulse of the heart is perceptible, the sounds are distant and feeble; there is no abnormal bruit; the urine is pretty copious, of smoke-brown colour, specific gravity 1010; it gives a cloudy precipitate on the application of heat, or on the addition of nitric acid; appetite pretty good; some thirst; tongue clean; bowels open. Cupped in the thorax to sixteen ounces. To have a sixth of a grain of potassio-tartrate of antimony, six grains of nitrate of potash, and an ounce of water, every five hours.

Urea was found to exist in the blood and saliva, by the use of the common salt test.

April 20. The heart's action is much more natural, the sounds clearer, the impulse is feeble; breathing easier; cough less troublesome; the face is less puffed; the quantity of urine is about three pints in twenty-four hours. Add to each draught a twelfth of a grain of tartar-emetic. To have a hot air bath.

22. The breathing is easier; the countenance much improved; expectoration less frothy; sounds of the heart clearer; appetite good; less œdema of the legs; urine less cloudy; pulse 105; tongue whitish. Continue the medicine. To have beef-tea. The urine is very acid. Nitric acid and heat give a cloudy precipitate.

23. Perspired a good deal during the night; no nausea or vomiting.

24. Pulse 108, soft, rather fuller; respiration 30; perspired for about two hours during the night; the perspiration was preceded by heat of skin and feverishness; the skin is now quite cool; bowels regular; specific gravity of urine a little below 1010; second sound of the heart very feeble.

25. The heart's impulse is heard over a more extended surface than natural. To have twelve grains of Dover's powder, and eight grains of nitrate of potash at bedtime; to have a quarter of a grain of elaterium early in the morning, or at noon.

27. Slept well, and perspired profusely about one o'clock in the morning, for about an hour, all over the body, but so much about his head as to wet his nightcap; he perspires now a little whenever he goes to sleep; skin cool; bowels confined, having been unacted upon by the elaterium; expectoration less. To have half a grain of elaterium twice a day.

28. Swelling of the legs much reduced; heart's sound more natural, and second sound louder: pulse 100, feeble.

29. There is a decided diminution of the precipitate in the urine, both by heat and nitric acid. The precipitate by nitric acid is small and cloudy, and floats on the surface of the fluid. Urine increased in quantity, specific gravity 1015; anasarca diminished; bowels slightly opened twice a day; tongue clean. To have a mutton chop. Ordered to take half a grain of elaterium three times a day.

May 1. Specific gravity of urine 1013; the elaterium made him sick, and purged him; anasarca and ascites diminished; pulse 104; tongue clean and moist: appetite good; heart's sounds louder. To have half a pint of porter.

3. Perspired a little in the night; anasarca nearly gone. Go on with the elaterium.

4. The skin of the legs is less scurfy, and the œdema much diminished. Perspired freely for about two hours in the night all over the body; heart's sounds clearer and more distinct. The impulse is scarcely perceptible to the hand, but the sounds, with this exception, are normal. Pulse 100, weak; abdomen less swollen, and fluctuation less distinct; urine more natural in colour; the precipitate is very much diminished in quantity; specific gravity 1015. Repeat the hot-air bath.

8. Last night he had a violent pain in his right shoulder, which continues, but is not so severe; specific gravity of urine 1020; deposit of albumen less; ascites less, but not completely gone. Omit the elaterium. Repeat the hot-air bath at night.

11. Anasarca nearly gone.

18. Had a bath last night, and perspired copiously; pulse 108; swelling quite gone, and he feels much stronger; specific gravity of urine 1017; the addition of nitric acid produces a slight cloudiness; bowels confined.

June 3. Is convalescent; no trace of urea in the saliva. Discharged, apparently in good health.

H. L., aged 52, a widow, charwoman, was admitted into Augusta ward, under the care of Dr. Todd, on the 24th of June, 1841. She is of a sallow complexion; never had any children. She has usually enjoyed good health. About six weeks ago she was seized suddenly, after being much fatigued, with pain in the left side, and shortness of breath, with a swelling of the whole of the upper part of the body. For these symptoms she was bled the next day, and took some purgative medicine, which she says made her mouth sore. About four days after this the swelling left the body, and went into the thighs and legs. The pain in the side was very severe, and continued for four or five days, when it gradually became better. She was at that time much troubled with palpitation, but she has not felt it lately; her strength has been much reduced, and she has lost flesh considerably. Last week she began to have a pain shooting up the right side of the head, which still continues. At present the pain in the head is still severe, and there is slight puffiness of the face. There is no swelling of the arms and body. Both legs and thighs are anasarcous; and there is considerable hardness of the cellular tissue on the backs of the feet, particularly in the right foot. She has no pain, with the exception of that in the head.

The urine is of a smoke brown colour, and with heat and nitric acid throws down albumen in considerable quantity; specific gravity 1010; bowels open; skin cool; tongue clean and moist; pulse 70; heart's action natural; no difficulty of breathing; chest sounds well on percussion; catamenia ceased three years ago; appetite good. To have a hot-air bath every night at bedtime, and to take five grains of Dover's powder and five of nitrate of potash three times a day. To be placed on middle diet.

June 26. To have a hot-air bath. The saliva and blood are found to contain urea when tested by the muriate of soda.

27. She perspired freely after the bath yesterday, and felt relieved by it. The pain in the head continues; the swelling is unaltered; bowels confined; pulse 80. Repeat the hot-air bath to-morrow. To have half a drachm of the compound jalap powder to-morrow morning.

29. Had an attack of diarrhœa yesterday, and therefore did not take the powder. Had the hot-air bath repeated yesterday. The swelling is a little reduced. To have an ounce of the compound chalk mixture every three hours.

July 1. The diarrhœa has ceased; the urine is altered in colour, and is now of a pinkish hue; the swelling of the legs is much reduced. Omit the chalk mixture. Continue the use of the powders. Repeat the hot-air bath.

2. The urine is of a light pinkish hue; the quantity of albumen is slightly diminished; the colour is not altered by nitric acid; specific gravity 1011.

8. The ammonia is much reduced, and she expresses herself improved. Bowels open; appetite better; urine of a smoke brown colour, contains a large quantity of albumen. Add a sixteenth of a grain of tartar emetic to each powder.

13. Complained yesterday of pain in the bowels: she took a dose of castor-oil, which opened the bowels freely, and relieved the pain.

15. The bowels are relaxed, and she has griping pains in the belly, and some tenesmus. Omit the tartar emetic, and apply eight leeches to the epigastrium.

Aug. 3. No swelling in the legs, but complains of pain in them. Countenance much improved; specific gravity of urine 1012; albumen much diminished. To have ten grains of Dover's powder at bedtime.

18. Discharged, very much improved.

During the last fortnight she has complained very much of pains in her limbs, for which she uses the hot-water bath, instead of the hot-air. Her aspect is much improved, her pains relieved, and the urine natural in appearance; the swelling of the legs entirely gone; appetite good; no traces of urea could be discovered in the saliva or the blood.—*Ibid.*

---

*Lacerated wound of the Arm, involving the Elbow-Joint—Question of Excision of the Joint or Amputation—Clinical Remarks by Mr. Ferguson.*—William Potts, aged 16, was admitted under Mr. Fergusson, on the 6th of August, at half-past five, P. M., in consequence of a severe injury of his right arm, produced by the machinery of a printing-press.

On examination, an extensive division of the integuments was found on the posterior part of the elbow, through which the articular surfaces of the bones were visible. The external condyle of the humerus was broken off, and lay loose in the wound; the olecranon process of the ulna was broken away, and the point of the coronoid process was also driven off. There were marks of contusion on the forearm and arm, and the textures in the immediate vicinity of the joint were severely injured.

Mr. Fergusson saw him soon after his admission, and, after a careful examination of the parts, considered amputation the only resource, as any attempt to save the limb seemed inadmissible, and likely to be attended with more danger to life than the removal of the member. Amputation was accordingly performed by the double-flap operation, opposite the insertion of the deltoid. The edges of the wound were held together with stitches; union by the first intention took place, and nothing unusual happened in the progress of the cure.

The limb was again inspected after the operation, and in addition to the injuries previously observed, the muscles in the forearm and arm were found severely contused, and blood was extremely extravasated among their fibres, and in all the surrounding textures: the humeral artery was apparently uninjured, but the ulnar nerve was denuded, contused, and seemingly overstretched.

In his subsequent observations on this case, Mr. Fergusson stated that there were several circumstances of considerable importance in its history, which he thought worthy the attention of the pupils. There were three methods of practice which he had had the option of following, viz., cleansing and dressing the wound, placing the parts in a favourable position, and leaving the case, in a great measure, to nature; performing excision of the ends of the bones, leaving clean cut surfaces instead of contused and lacerated, as presented by the wound; or the total removal of the parts above the seat of injury. The first of these plans he should most willingly have preferred, had he entertained any hope of being able to have saved the limb; but the compound comminuted fracture, compound dislocation, and severe injury to all the textures in the vicinity, seemed to preclude any hope of this kind: the latter circumstance induced him to reject the method of excision; for, though the wound in the skin and the condition of the bones seemed favourable to this practice, and it might have been exceedingly easy to remove the injured ends of the bones, he did not deem the case, in other respects, a proper one for this operation, an operation which he thought too valuable in practice to be carelessly injured in character by the selection of a very questionable case for its performance. He had seen the practice in question adopted in a somewhat similar instance, in which, however, the destruction of parts seemed less extensive; and here, as in various other cases which had came under his notice, the result had been such as to induce him to recommend caution as to the indiscriminate performance of such an operation.

Amputation, under all the circumstances, seemed the most advisable proceeding, and the dissection of the limb clearly evinced its propriety.

Mr. Fergusson also directed attention to what he considered a practical matter of great consequence in this operation. To the surprise of several of the pupils, the limb was divided much higher up than seemed to them necessary, as the skin for several inches lower down appeared sound and uninjured; but, he observed, that though the skin gave no indications of the extent of the injury to the textures which it covered, the muscles, nevertheless, displayed various marks of contusion, such as ecchymosed spots in different parts, even as high up as the seat of amputation. He imagined that in such a case as this; when amputation was done so soon after the injury, there was not sufficient time for the integuments becoming much discoloured, or evincing any alarming appearance; yet this case afforded a good opportunity of enforcing the rule of practice he had pursued; for though during the operation he had removed a small portion of muscle on one of the flaps which appeared to him in a suspicious condition, another smaller portion in a like state, which he had left, had actually sloughed; the slough, however, being so small, that it was correct enough to state, in the usual sense of the term, that the stump had healed by the first intention.

His object in alluding to this point, was to caution against the practice of amputating in the immediate vicinity of such a wound, as he had repeatedly seen sloughing ensue to such an extent as to leave stumps by no means creditable to good surgery.

In this instance Mr. Fergusson used threads of caoutchouc in making the sutures, and stated that his experience in the use of this material had not yet been such as to induce him to give an opinion of its value: he wished the pupils to watch for themselves the trials he was making of it in different cases at present in the hospital.—*Ibid.*

---

*Schirrous Pylorus--death--Necrotomy.* By Mr. William Proctor. Clinical Assistant, Westminster Hospital.—Mary Shaw, æt. 43, was admitted, 15th June, 1841, into Westminster Hospital, under the care of Dr. Burne. Until three yeare since she enjoyed good health; at that time she had a severe attack of scarlatina, with low fever: the debility consequent on this was lengthened by a cold taken during recovery. Shortly after complete convalescence she began to feel pain and uneasiness in the stomach, after taking food, followed by nausea and heartburn; which symptoms slowly increasing, tenderness in the epigastrim supervened; and as the disease increased in severity and advanced, every thing taken into the stomach was rejected some little time afterwards; vomiting took place at intervals (without any apparent exciting cause) of matters having different appearances; sometimes a green mucus; at others, that peculiar fluid like coffee grounds, often mixed with undigested food. She was not able to take any thing solid—sago, arrow-root, and nutritive articles of that kind, constituted her diet; but even these she was able to bear only in small quantities. In this state she applied to Dr. Richardson, who, having assured himself of the nature of her disease, sent her into the hospital.

At the time of her admission she was extremely emaciated: the eyes sunk, the cheekbones prominent, and the whole face lean and linear; the skin was dry, and imparted a rough sensation to the touch; the tongue was dry and furred; the bowels (as they had been all along) very costive: pulse 70, small as a thread, and feeble. She vomited usually about an hour after taking food; and attacks of vomiting, more particularly during the night, still recurred; the matter brought up, being a dark-coloured fluid, generally mixed with matters previously taken into the stomach. Neither at this time, or at any other, was gastric pain experienced; nothing beyond an occasional and disagreeable feeling of distension.

On inspecting the abdomen on several occasions, the outline of the stomach (being then distended) was visible and distinctly traceable; the thin abdominal parietes being elevated and bulging, in the shape of the stomach, across the epigastric and umbilical regions. Placing the hand on this latter region, to the right of the linea alba, a tumor, the size of half an orange, was clearly palpable, and more or less moveable; its situation was found to vary in some degree, according as the stomach was distended or contracted.

The question that organic disease existed being indisputable, means were employed to allay the vomiting, which was at this time the most urgent symptom; and to support the strength, as far as possible, by diet of milk and sago, with beef-tea, taken in small quantities, and frequently, in order that the stomach might not be suddenly overloaded. The bowels were regulated by common enemata; and as a palliative, she was ordered—

Mistur. Creasoti, ℥j. t. d.

28th.—She has been pursuing the above treatment up to this time; and though there is less vomiting and uneasiness of the stomach, no decided improvement is perceptible; indeed she appears to grow weaker daily. Dr. Burne ordered the mixture to be continued, and an enema of milk every morning, and of beef-tea, egg, and flour, twice a day, with a view to nourish the patient.

July 14—Has continued the preceding plan, with some benefit; is no weaker than on her admission; vomiting on the whole less, though more frequently, and often in the night.

20th.—The stomach continues more settled; she is able to pursue the plan prescribed by Dr. Burne, and to take a light pudding daily; notwithstanding this, no decided improvement is perceptible. The vomiting returns if the bowels are not open, and emaciation goes on.

28th.—Is gradually getting weaker, and there is more emaciation. Great care being observed, the stomach is induced to retain her food. The attacks of vomiting return frequently during the night, and soon after taking any solid substance, as bread, which was once or twice attempted. She requires the use of aperient (in addition to the nutritive) enemata, in order to relieve the bowels.

Aug. 14th.—Soon after the last report her memory began to fail, and she appeared lost when asked a question. Is much worse in all respects, being able to take scarcely any food; fluid, like coffee grounds, continues to be vomited. For the last three days the bladder required emptying by the catheter; violent hiccough supervened. Her remaining powers failed rapidly, and in the course of the evening she expired.

*Post-mortem examination thirty hours after death.*—The whole body emaciated, and attenuated to the utmost degree. The abdominal parietes being reflected, a tumor, the size of a small apple, rather flattened, was seen occupying the pyloric extremity of the stomach, and lying in the umbilical region. The position of the stomach was somewhat oblique; the lesser curvature looking to the right side, and passing transversely downwards from the left hypochondiiac to the above named region. The stomach itself was unnaturally large, capable of holding five pints. On laying it open, the cardiac parietes were thin; the mucous membrane soft, and marked with two or three particles of minute dotted vascularity. The tumor completely encircled the pylorus; and, on cutting through it, a rough grating sensation was communicated to the scalpel; the peritoneal coat around it was entire, but involved in the general thickening. Internal to this the tumor was pale, and its extent well defined by a hard white line of cartilaginous consistence. The proper schirrosity occupied the place of the muscular and submucous tissues: this non-analogous structure, extending all round the pylorus, was more than half an inch thick, of a yellowish-white colour, intersected with dense white lines of a fibrous aspect; the interstices filled up with a whitish hard concrete substance. The pylorus itself was diminished in size so as barely to allow the passage of a quill within it; in several places the mucous membrane was ulcerated. The duodenum was of natural calibre, and otherwise in a healthy state. The liver was atrophied, but healthy in structure. The omentum appeared of increased vascularity.—The other viscera were quite healthy; and no schirrous induration occupied the intestinal canal —*Med. Gazette.*

---

*Amaurosis cured by Iodide of Potassium.*—H. Charlwood, aged 13 years, was admitted into the Middlesex Hospital, January 19, under the care of Mr. Arnott. He cannot distinguish, with his left eye, any object, and so complete is the loss of perception in this organ, that it is only where there is a bright light that he can distinguish the shade occasioned by the passage of the hand between it and the light. This pupil is more dilated than that of the right eye; and the iris, without being quite fixed, has but a very limited extent of motion, and that is sluggish. The vision of the right eye is good. The boy has a pallid appearance. He says that he has often been affected with pain in the head and the left side of the face. About two years ago he first noticed that when he looked at objects there appeared a little black spot, as it were, before the left eye, which partially obscured the object. This spot gradually increased in size until about three months since, when vision of the left eye was completely lost. On examining his mouth the teeth are found to be very irregular and crowded, owing to his not having shed any of his temporary molars or canines, some of which are decaying. He has occasionally had toothache. Some time ago a portion of bone came away from his upper jaw, and he pointed to the palate as the spot whence it separated, and where there is the appearance of a cicatrix.

Mr. Arnott remarked, that on inquiry into the causes of amaurosis in this case, with a view to determine the plan of treatment to be adopted, it was first of all clear that there was no evidence of local excitement, and that it was not a case requiring, and not likely to be benefitted by, mercury or depletion. It was probable, in the next place, that the amaurosis might be connected with the diseased condition of the upper jaw which had previously existed, and possibly did still exist, and the irritation from the presence, at his age, of temporary and permanent teeth at the same time. But, as the tongue was coated, it was advisable to get his stomach and bowels first into a freer state. Accordingly, until the 30th of the month, he underwent a course of alteratives and aperients, but without benefit to the sight.

Feb. 1. Eight temporary teeth and stumps were extracted; namely, one canine and three molars from the upper jaw on the left side, one molar on the right, and three molars from the lower jaw.

Feb. 6 and 8. No improvement; sees no better.

It was now determined, with a view to the possibly still existing diseased state of the bone or periosteum of the jaw, to put him upon a course of iodide of potassium and sarsaparilla. He was, therefore, ordered on the 8th to have a grain and a half of iodide of potassium, with two ounces of decoction of sarsaparilla, three times a day.

15. There has been some improvement in the left eye during the last day or two; on trial this afternoon, he distinguished when the hand was held up before him whether it was open or closed, and he discovered that three fingers only were extended. Continue the medicine.

22. A still further improvement; he says there is less thickness of vision.

27. Is slightly better; grinds his teeth at night. To have a scruple of compound jalap powder directly. Continue the medicine.

March 8. Distinctly better. Distinguished a black button on the grey ground of a waistcoat of one of the pupils.

11. Distinguished the colour of a sovereign from that of a shilling.

15. Recognised the colour of a blue flower in the button-hole of a gentleman's coat.—Told the time by Mr. Arnott's watch after examining it for a little time, and holding it in various positions to the light. Has had soup diet; to now have meat. Continues his medicine.

April 1. Sight improving; can read the diet-card, but he complains of pain in his head to-day. Let his meat be changed for soup. Stop his medicine for a day or two, and let him have a compound senna draught directly.

8. Read, though with difficulty, in one of the religious tracts given by the clergyman to the patients. To go on with the iodide and the sarsaparilla. The pupil of the left eye is now of nearly the same size as that of the other, and the motions of the iris are almost as rapid.

May 4. The boy has for some time been able to read his Bible with facility. Discharged cured.—*Lancet.*

It seems iodide of potassium cures many diseases. It may be that we are running too hard upon the preparations of iodide, but it is certain that the constitutional affections which resist the action of ordinary remedies, yield nearly as readily to iodide as to mercury, and that, in some cases, the constitutional disturbance is less, and the action of the remedy at least as prompt. But we object decidedly to the large doses in which these remedies have been given of late: not that they are always dangerous, but that in some cases mischievous results follow. Besides, we think the whole system is best revolutionized by minute doses.

---

*Extraordinary Developement of the Kidneys in a new-born Infant.* By DR. F. OESTERLEN, of Murrhardt.—The great size of the abdomen of the infant formed a serious obstacle to its delivery; the head and breast passed the vulva easily, but it required considerable efforts on the part both of mother and accoucheur to deliver the abdomen. The child was dead, and the enlargement of the abdomen was found to depend on an enormons developement of the kidneys, which had displaced the liver and all the other abdominal viscera. They were converted into a vesicular structure, and the granular portions were considerably enlarged. The vesicles were regarded as true hydatids, into which the parenchyma of the kidneys had been transformed.

This disease, though rare in the young of the human subject, is not at all uncommon in lambs.—*Ibid., from Neue Zeitschrift fur Geburtskunde.*

---

*Case of general Emphysema caused by an Ulcer of the Larynx.* By M. BODDAUT.—A girl about 15 years of age, had for some time been affected with pain in the throat, difficulty in swallowing, and general ill health. On the 20th of March, the pain of the throat had become very severe; she could not swallow any solid food, and the jaws could not be separated but with difficulty. On the night of the 22d, emphysematous swelling appeared on the neck and the right side of the face, and as the girl was much frightened, and cried abundantly, the swelling rapidly increased over the other parts of the body. By the 24th, the emphysematous swelling had involved the whole of he face, neck, trunk, and upper extremities, but had not extended to the lower; the palms of the hands being alone exempt from it. She died on the night of the 24th.

No lesion was observed in the lungs. The right ventricle of the heart was gorged with frothy blood. When the larynx and trachea were laid open, a small round ulcer, pierced in its centre by an opening similar to what a lead drop would have produced, was noticed in the right ventricle of the larynx, a little below the vocal cord. Four or five superficial, but not distinctly circumscribed ulcers were observed in the pharynx.—*Ibid., from Annates de la Societé de Medicin de Gand.* August 1840.

